# 

**Published:** 1825-01-01

**Authors:** 


### Contents

OF

### No. 3. Vol. II. (NEW SERIES) for JANUARY, 1825.

[*No.* 19, *ANALYTICAL SERIES.*]

Art. I.

FEVER—CONTAGION—QUARANTINE.

1. Dr Burnett's Official Report on Fever &c. .. .. ..
2. Dr. Mills on Fever and Inflammatory Complaints .. ..
3. Dr. Maclean on Quarantine and Contagion .. .. ..
4. Mr. Wade's Observations on Fever .. .. .. ..
5. Dr. Stoker's Medical Reports of the Fever Hospital .. — 1

II.

Mr. Suttleffe's Medical and Surgical Cases, the result of 38 years' practice .. .. .. .. .. .. .. .. .. .. 23

III.

ANATOMY, PHYSIOLOGY, AND PATHOLOGY OF THE SKIN.

1. Mr. Chevalier's Lectures on the Anatomy and Functions of the Skin .. .. .. .. .. .. .. .. .. ..
2. Dr. J. L. Alibert, on the Diseases of the Skin .. ..
3. Mr. Plumbe's Practical Treatise on Diseases of the Skin. — 29

IV.

Essays on Various Subjects of Medical Science. By David Hosack, M.D. F.R.S. .. .. .. .. .. .. .. .. 53

V.

Dr. John Forbes on the Use of the Stethoscope and Percussion in the Diagnosis of Diseases of the Chest, with Original Cases and Dissections .. .. .. .. .. .. .. .. 61

VI.

Dr. Alison's Observations on the Pathology of Scrofulous Diseases, with a View to their Prevention .. .. .. .. 98

### VII.

An Essay on Curvatures and Diseases of the Spine, by R. W. BAMPFIELD, Esq. &c. .. .. .. .. .. .. .. 112

### VIII.

### Quarterly Periscope

OF

### PRACTICAL MEDICINE;

BEING

*THE SPIRIT OF THE MEDICAL JOURNALS,*

FOREIGN AND DOMESTIC.

### I. PHYSIOLOGY.

1. Dr Graves on Free Acid in the Human Stomach .. .. 145

2. Dr. Hamilton's Instances of Panic producing Fever .. .. 148

3. Curious Physiological Effects of Lightning .. .. .. 149

4. M. Bellingeri on the Spinal Marrow and Nerves .. .. 151

5. Mr. Arnott on the Physiological Effects of Ptyalism .. .. 153

### II. PATHOLOGY.

1. Messrs. Cullen and Carswell on Melanosis .. .. .. .. 154

2. Dr. Craigie on Biliary Calculus .. .. .. .. .. .. .. 159

3. Dr Carter on Occult Disease of the Chest .. .. .. .. 161

4. Dr. Duncan on Fatal Inflammation of Veins. .. .. .. 161

5. Post-mortem Examination of Lord Byron .. .. .. .. 164

6. Dr. Martinet on Inflammation of the Nerves .. .. .. 165

7. Dr. Gairdner on Perforations of the Alimentary Canal in Children .. .. .. .. .. .. .. .. .. .. 170

8. M. Louis on a peculiar Species of Gastritis . .. .. .. 173

9. M. Bourdon on certain Organic Diseases of the Stomach .. 174

10. Mr. Callow's Case of Abscess in the Parietes of the Stomach 180

11. Dr. Meyers on Cynanche Trachealis .. .. .. .. .. .. 181

12. Legallois' Case of Perforated Intestine .. .. .. .. 182

13. Dr. Teeling on Suppression of Urine .. .. .. .. .. .. 183

14. Sir J. Moriarty on Hepatic Phthisis .. .. .. .. .. .. 184

15. M. Bayle on various Forms of Anomalous Gout.. .. .. 187

16. Remarkable Case of Acute Cerebral Affection .. .. .. 192

17. Mr. Carmichael on Tetanus.. .. .. .. .. .. .. .. .. 193

18. Dr. Gairdner on Purpura Hæmorrhagica .. .. .. .. 194

19. Dr. Crampton on Conversion of Disease .. .. .. .. 197

20. Dr. Macauley on the Effects of Lightning .. .. .. .. 199

21. Dr. Vilermay on Inflammation of the Appendix Cœci .. 200

22. Curious Instance of Partial Intermittent Fever .. .. .. 201

23. Mr. Hamilton's Cases of fatal Fever from opening a Grave . 202
24. Dr. Reid on the Pathology of Epilepsy .. .. .. .. 205
25. Dr. Brooke on Liver Cough .. .. .. .. .. .. 208

III. SURGERY.

1. Amputation of the Lower Jaw, by Dr. M'Clelland .. .. 210

2. Drs. Kinglake and Balfour on the Treatment of Hernia .. 211

3. Dr. Wedemeyer on Paracentesis Thoracis .. .. .. .. 213

4. Mr. Hume's Case of Tracheotomy in Cynanche Trachealis.. 215

5. Cases of Extirpation of the Ovaria .. .. .. .. .. 215

6. Dr. Belcher on Chronic Enlargement of the Tonsils .. .. 217

7. Sudden Death during an Operation .. .. .. .. .. 218

8. Great Tumour removed from the Neck, by M. Dupuytren.. 219

9. Mr. Isaac Ryall on Purulent Ophthalmia of Infants .. .. 220

10. Laryngitis, Tracheotomy in. by Mr. Carmichael .. .. .. 223

11. Mr. A. C. Hutchison's Case of Hæmor hage into the Bladder 224

12. Delpech's successful Amputation at the Hip-Joint .. .. 225

IV. THERAPEUTICS.

1 Drs. Kolley and Zink on the use of Iodine .. .. .. .. 229

2. Dangers of Iodine.. .. .. .. .. .. .. .. .. .. .. 233

3. Dr. O'Halloran on the Treatment of Dysentery .. .. .. 234

4. Dr. Balfour's *striking* Cures.. .. .. .. .. .. .. .. 234

5. Dr. Crampton's Case of Poisoning by Opium .. .. .. 235

6. Dr. O'Halloran on the Treatment of Pneumonia .. .. .. 236

7. Dr. Sharkey on the Treatment of Diabetes .. .. .. .. 237

8 Dr. O'Brien on the Use of Sulphate of Quinine in Tyhhus .. 237

9. Hospital Reports from Laennec, in La Charité

   1. On Fevers .. .. .. .. .. .. .. .. .. .. 240

   2. On Pulmonic Inflammations .. .. .. .. .. .. 241

   3. On Phthisis .. .. .. .. .. .. .. .. .. 241

   4. On Diseases of the Heart .. .. .. .. .. .. .. 241

   5. On Chronic Peritonitis .. .. .. .. .. .. .. 242

   6. On Sciatica and Neuralgia.. .. .. .. .. .. .. 242

V. MIDWIFERY.

1. Dr. Payne on Puerperal Fever, with Commentaries .. .. 243
2. Mr. Sanders on Puerperal Fever .. .. .. .. .. .. .. 244

VI. MEDICAL JURISPRUDENCE.

1. Mr. Greenhow on Medical Remuneration, and a Plan for its Improvement .. .. .. .. .. .. .. .. .. .. 246

IX.

ANALECTA MINORA, OF LITTLE PERISCOPE.

1. Mr. Amesbury on the Preservation of Dead Subjects .. .. 248
2. Emplasto-dermic Treatment of Diseases .. .. .. .. 249

3. Contagious Glanders in Horses .. .. .. .. .. .. 249
4. Corrector of Cancerous Fœtor .. .. .. .. .. .. 249
5. Non-Union of Fractures .. .. .. .. .. .. .. 249
6. Substitute for Castor Oil .. .. .. .. .. .. .. 249
7. Congestive Fever in the Sea .. .. .. .. .. .. .. 249
8. Preservation of the Stomach by Poison .. .. .. .. 250
9. Pathology of a Pinch of Snuff .. .. .. .. .. .. .. 250
10. Phrenology .. .. .. .. .. .. .. .. .. .. .. 251

X.

Bibliographical Record .. .. .. .. .. .. .. .. .. 251

XI.

OBITUARY.

1. Dr. Cleverly .. .. .. .. .. .. .. .. .. .. .. 256
2. Mr. Parker .. .. .. .. .. .. .. .. .. .. .. 257
3. Mr. O'Beirne .. .. .. .. .. .. .. .. .. .. 257
4. Dr Bridger .. .. .. .. .. .. .. .. .. .. .. 258

XII.

INTELLIGENCE, &c.

1. Lithotomy, Mr. Weiss's new Lithontriptor .. .. .. .. 258
2. Valuable Collection of Books on Natural History, &c .. .. 258
3. Mr. Curtis's Lectures on Diseases of the Ear .. .. .. 259

XIII.

EXTRA-LIMITES.

I. Philo-Medicus on Self-Advertising, and on the Comparative Importance of Certain Medical Degrees.. .. .. .. 260

II. Mr. Hickes on Pregnancy complicated with Ascites .. .. 265

Additional Cases of the same.. .. .. .. .. .. .. 267

III. Retrospections

1. Divided Arteries .. .. .. .. .. .. .. .. 268

2. Fowler's Solution of Arsenic, an old formula .. .. 268

IV. The Surgeon and Apothecaries' Drug Company . .. .. 269

V. Explanation of Mr. Weiss's new Lithontriptor .. .. .. 270

VI. Mr. Seaman's Shampooing Baths .. .. .. .. .. .. 270

VII. Mr. Battley's Letter on Opium .. .. .. .. .. .. .. 273

XIV.

Correspondence, Additional Subscribers, &c.

